# Unseen Artificial Intelligence—Deep Learning Paradigm for Segmentation of Low Atherosclerotic Plaque in Carotid Ultrasound: A Multicenter Cardiovascular Study

**DOI:** 10.3390/diagnostics11122257

**Published:** 2021-12-02

**Authors:** Pankaj K. Jain, Neeraj Sharma, Luca Saba, Kosmas I. Paraskevas, Mandeep K. Kalra, Amer Johri, John R. Laird, Andrew N. Nicolaides, Jasjit S. Suri

**Affiliations:** 1 School of Biomedical Engineering, IIT (BHU), Varanasi 221005, India; pankajkrjain.rs.bme17@iitbhu.ac.in (P.K.J.); neeraj.bme@iitbhu.ac.in (N.S.); 2 Department of Radiology, Azienda Ospedaliero Universitaria (A.O.U.), 10015 Cagliari, Italy; lucasabamd@gmail.com; 3 Department of Vascular Surgery, Central Clinic of Athens, 14122 Athens, Greece; paraskevask@hotmail.com; 4 Department of Radiology, Massachusetts General Hospital, 55 Fruit Street, Boston, MA 02114, USA; MKALRA@mgh.harvard.edu; 5 Division of Cardiology, Queen’s University, Kingston, ON K7L 3N6, Canada; amerjohri@gmail.com; 6 Heart and Vascular Institute, Adventist Health St. Helena, St. Helena, CA 94574, USA; Lairdjr@ah.org; 7 Vascular Screening and Diagnostic Centre, University of Nicosia, Nicosia 1700, Cyprus; anicolaides1@gmail.com; 8 Stroke Diagnostic and Monitoring Division, AtheroPoint™, Roseville, CA 95661, USA

**Keywords:** Unseen AI, Seen AI, UNet deep learning, multi-ethnic studies, carotid atherosclerotic wall plaque

## Abstract

Background: The early detection of carotid wall plaque is recommended in the prevention of cardiovascular disease (CVD) in moderate-risk patients. Previous techniques for B-mode carotid atherosclerotic wall plaque segmentation used artificial intelligence (AI) methods on monoethnic databases, where training and testing are from the “same” ethnic group (“Seen AI”). Therefore, the versatility of the system is questionable. This is the first study of its kind that uses the “Unseen AI” paradigm where training and testing are from “different” ethnic groups. We hypothesized that deep learning (DL) models should perform in 10% proximity between “Unseen AI” and “Seen AI”. Methodology: Two cohorts from multi-ethnic groups (330 Japanese and 300 Hong Kong (HK)) were used for the validation of our hypothesis. We used a four-layered UNet architecture for the segmentation of the atherosclerotic wall with low plaque. “Unseen AI” (training: Japanese, testing: HK or vice versa) and “Seen AI” experiments (single ethnicity or mixed ethnicity) were performed. Evaluation was conducted by measuring the wall plaque area. Statistical tests were conducted for its stability and reliability. Results: When using the UNet DL architecture, the “Unseen AI” pair one (Training: 330 Japanese and Testing: 300 HK), the mean accuracy, dice-similarity, and correlation-coefficient were 98.55, 78.38, and 0.80 (*p* < 0.0001), respectively, while for “Unseen AI” pair two (Training: 300 HK and Testing: 330 Japanese), these were 98.67, 82.49, and 0.87 (*p* < 0.0001), respectively. Using “Seen AI”, the same parameters were 99.01, 86.89 and 0.92 (*p* < 0.0001), respectively. Conclusion: We demonstrated that “Unseen AI” was in close proximity (<10%) to “Seen AI”, validating our DL model for low atherosclerotic wall plaque segmentation. The online system runs < 1 s.

## 1. Introduction

### 1.1. Stroke Statistics, Causes of Stroke, and Need for Screening

Stroke is the third leading cause of death in the modern age. As per the 2018 data from the Centre for Disease Control and Prevention (CDC), one in every six deaths from cardiovascular diseases (CVD) is from stroke [1]. In the USA, every 40 s, someone suffers from a stroke, and every 4 min, someone dies of stroke [1]. The primary cause of stroke is the formation of atherosclerosis disease in carotid arteries [2], where the plaque is formed in the lumen–intima and media layers [3]. The LDL penetration in the arterial walls accelerate the plaque formation, such as fibrosis, fibrin, and macrophages due to a sedentary lifestyle [3]. This plaque then ruptures over time, causing embolism in the brain leading to stroke [2,4]. The plaque formation worsens with comorbidities such as diabetes [5], hypertension [6], renal disease [7], and heart disease [8]. Thus, it is vital to detect wall plaque during the early stages of its formation using angiography screening techniques [9,10,11].

### 1.2. Importance of Imaging Modalities and Plaque Quantification

Various imaging modalities such as magnetic resonance imaging (MRI) [9], computed tomography (CT) [12], and ultrasound (US) [5,13] are used for the screening of the disease. The US is cheap, user-friendly, has a smaller foot print, is non-invasive, and does not use a radiation-based method [14]. Therefore, B-mode ultrasound scans are primarily used for the detection of atherosclerotic plaque in carotid arteries. These ultrasonic scans offer image-based phenotypes as leading biomarkers for stroke risk assessment such as carotid intima–media thickness (cIMT) [15], intima–media thickness variability (IMTV) [16,17], maximum plaque height (MPH) [18], total plaque area (TPA) [19,20,21], total plaque volume (TPV), and lumen diameter (LD) [22,23,24]. Various automated and semi-automated methods have been proposed to calculate imaging biomarkers in the past [25]. These biomarkers have their own relevance based on the study objective involved [26]. The measurement of the plaque area was proposed as an alternate method of stroke risk prediction [19]. The carotid plaque area could be manually delineated between lumen–intima (LI) and media–adventitia (MA) borders, but this is tedious, error-prone, time-consuming, and leads to inter-observer variabilities [27,28]. Thus, one needs to automatically estimate the plaque wall area [29,30]. All the above methods involve conventional and statistical image processing methods for the LI and MA borders’ segmentation and quantification.

### 1.3. Brief Background of AI Literature

Artificial intelligence (AI)-based methods are becoming more popular in the healthcare industry for automated diagnosis and prognosis [31,32]. Using the AI methods, researchers contributed some ad hoc methods involving machine learning methods for stroke risk assessment. Suri and his group significantly contributed to research in stroke risk assessment using machine learning [33,34,35,36]. These machine learning methods are not entirely automated as these involve human intervention for the extraction of plaque features from the region of interest (ROI) area.

Deep learning (DL) techniques which are also part of AI have become more popular in healthcare imaging in recent years [37,38,39,40,41,42]. Deep learning techniques involve less human intervention and rely on directly extracting features from the images [43]. Several studies have been attempted recently using DL in medical imaging [44,45,46,47]. However, the current DL models use training and testing databases from the same ethnic group or cohort in recent studies. The database from one ethnic group or US scanner was is partitioned into training and testing pools. Therefore, the system only learns from the images coming from one ethnic group or US scanners. Thus, in a macro view, there are chances that the system is biased towards the images from one ethnic group or US scanner [48]. This view lays the foundation of the hypothesis of our research work. A few studies involve assessing CVD risk, but their focus was solely on enhancing the data size rather than studying the system’s performance using a multi-ethnic database [49,50].

### 1.4. Motivation, Hypothesis of Unseen AI, and Concept of Global Segmentation System

**Figure 1:**
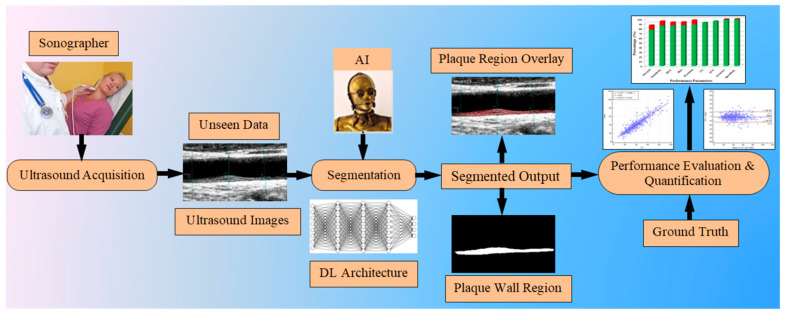
System diagram for unseen data processing.

We hypothesized that the DL model will be unbiased when we use separate training and testing databases. This means when we train the system with one ethnic group or cohort, it will be treated as a Unseen AI model for other ethnic groups or cohorts and vice versa. Therefore, we proposed two Unseen AI models which involve training the Japanese (Jap) cohort and testing on Hong Kong (HK) data and vice versa. We compared our Unseen AI model with the Seen AI model to validate our hypothesis that the Unseen AI systems are performing their function. We combined two ethnic data and made a pool of mixed data. This mixed database was used as the input to the Seen AI model. In the Seen AI model, we cross-validated each image using 10-fold cross-validations. Thus, in the Seen AI model, the cohort type was known to the system. Furthermore, we can also check the performance of the Unseen AI model against the Seen AI model by cross-validating each cohort. Figure 1 shows the global system diagram for unseen image segmentation.

### 1.5. Layout of This Study

The architecture of this article includes six major sections. Section 1 describes the introduction, background, and hypothesis of the current work. The methodology section consists of patient’s demographics, data collection and ground-truth data preparation, UNet-based deep learning architecture, and experimental protocols. All these sub-sections are described in Section 2. Section 3 contains the results of all experiments described in the methodology section. Section 4 describes the performance evaluation section where each performance tests results and their corresponding graphs are described. Finally, Section 5 and Section 6 contain the discussion and conclusion.

## 2. Methodology

To prove our hypothesis via our experiments, the choice of the deep learning architecture and the image dataset is essential. We took special care in the architecture design and the multi-ethnic datasets, which were vital for the “Unseen AI” analysis and benchmarking against “Seen AI” standard protocols. Thus, the methodology section mainly consists of three parts: (1) patient demographics, data collection, and data preparation for multi-ethnic datasets; (2) DL architecture; and (3) the experimental protocol used for the “Unseen AI” and “Seen AI” experiments using the multi-ethnic databases.

### 2.1. Patient Demographics, Data Collection, and Data Preparation

#### 2.1.1. Patient Demographics for the First Group: Japanese Cohort

The Japanese cohort consisted of 330 of left and right common carotid arteries B-mode ultrasound images taken from 165 patients. The male/female proportion in the cohort was 127/38. All patients were examined retrospectively, and ethics approval was granted by the institutional review board (IRB) of Toho University, Japan. All patients were informed, and written consent was obtained before the examinations. The baseline characteristics of the Japanese diabetes patients included a mean age of 68.25 ± 11.23 years. The mean hemoglobin (HbA1c) of all patients was 6.22 ± 1.04 mg/dL, low density lipoprotein (LDL) cholesterol of 101.59 ± 31.03 (mg/dL), high density lipoprotein (HDL) cholesterol of 51.05 ± 14.56 (mg/dL), and total cholesterol (TC) of 175.39 ± 36.03 (mg/dL). Out of 165 patients, 73 patients were smokers, 19 had a family history of CVD and 116 had hypertension. The mean systolic and diastolic blood pressure values were 134.06 ± 9.14 (mm/Hg) and 87.03 ± 4.57 (mm/Hg). The mean eGFR value for the cohort was 47.31 ± 19.93 (mL/min/1.73 m^2^). This dataset was used in our previous studies [33,34,40].

#### 2.1.2. Patient Demographics for the Second Group: Hong Kong Cohort

A total of 300 images from 50 patients (6 images per patient, 3 scans at each side of neck showing three different acquisition scans: anterior, anterolateral, and posterolateral with simultaneous ECG gating). All subjects were postmenopausal Chinese women aged between 54 and 67 years (mean age 60.2 years). Subjects were informed and consent was obtained before their inclusion in study. In the pool of 50 females, 28 females were diagnosed with different diseases: one was diagnosed with diabetes, three with hypertension, seven were diagnosed with hypercholesterolemia, whereas 15 had both hypertension and hypercholesterolemia, and two had all three abnormalities. The rest controlled population of 22 females had normal blood pressure, total cholesterol, and glucose levels in fasting blood. These data were used in one of our previous studies [15].

#### 2.1.3. Data Acquisition and Ultrasound Imaging for the Two Ethnic Groups

Japanese Cohort: All Japanese ultrasound images were scanned by Aplio XV, Aplio XG, Xario, Toshiba, Inc., Tokyo, Japan Ultrasound scanner, equipped with a 7.5 MHz linear array transducer. An experienced sonographer performed all US scans. The timeline of data collection was between July 2009 and September 2010. The recommendations of the American Society of Echocardiography Carotid Intima–Media Thickness Task Force were followed during the acquisition of these images. In this database, the mean resolution was 0.052 ± 0.01 mm/pixel.

Hong Kong Cohort: The Hong Kong (HK) database was examined using Sonoline Antares (Siemens Medical Solutions, USA, Inc., Malvarn, PA, USA) ultrasound scanner equipped with a 13.5 MHz linear transducer. The sonographer digitally captured a 10 s clip of each scan for offline analysis. Similarly, six segments (3 left and 3 right) of the left and right carotid arteries were archived for each subject. These data were used in some of our previous studies [15,51].

#### 2.1.4. Ground-Truth Data Preparation

An image-processing expert trained by an experienced cardiologist prepared the ground-truth binary mask images. We used a clinically acceptable image tracing tool developed by Atheropoint™ LLC, Roseville, CA, USA, to trace the CCA images’ lumen–intima (LI) and media–adventitia (MA) borders. The region between the lumen–intima (LI) and media–adventitia (MA), i.e., the atherosclerotic wall plaque, generates binary masks for the DL system. We used PowerToys (Microsoft) software for resizing the raw images to a size of 224 × 224 × 3. Additionally, the corresponding binary mask was also resized to the same size as of raw images using same software.

### 2.2. UNet-Based Deep Learning Architecture

**Figure 2:**
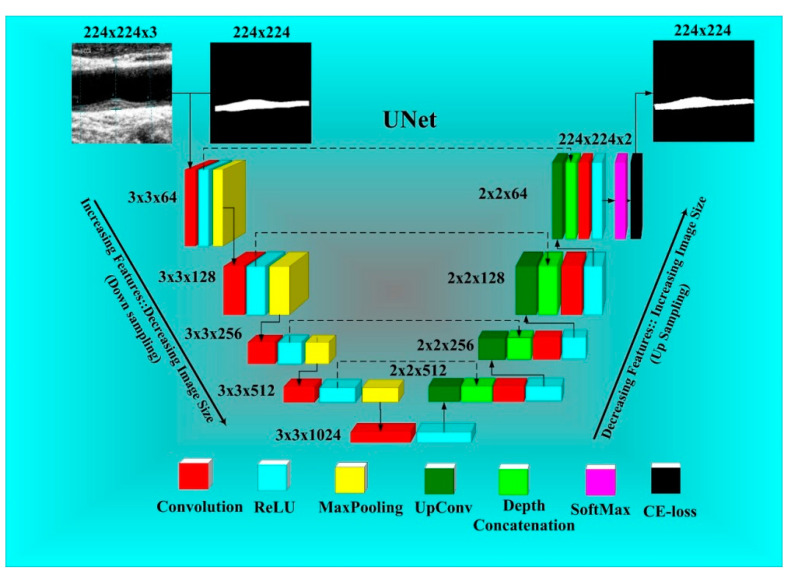
UNet: Four-layer DL architecture for atherosclerotic plaque wall segmentation.

A four-layer DL architecture UNet with a stack of four encoders and four decoder stages on both sides of the U-shape is shown in Figure 2. The encoder stages up-sample the images, while the decoder stages down-sample the images. Each encoder stage of UNet consists of a 2D-convolution layer (red) followed by ReLU (turquoise) and a MaxPooling layer (yellow). Similarly, each decoder stage consists of a stack of up-convolution-2D layers (dark green), depth-concatenation (light green), 2D-convolution (red) ReLU (turquoise) and a MaxPooling layer (yellow). A grayscale US image was given as an input of size 224 × 224 from the encoder stage 1. At stage one, the number of convolution filters was 64, which doubled in each next stage of the encoder module. Therefore, the numbers of filters in each stage become 128, 256, and 512. In contrast, the number of filters is reduced to half in each stage of the decoder. When counting from the bottom of the picture, these numbers are 512, 256, 128, and 64. Both the encoder and decoder modules are connected via a bridge network. The bridge network consists of 3 × 3 × 1024 filters. The bridge network provides a stack of features that are compatible with concatenating to the last encoder layer after downsampling from the first upsampling layer.

From each encoder stage, spatial features are extracted and transferred to the downsampling layer at the corresponding level of depth via a skip connection. Furthermore, these features are added with the features of the previous decoder or bridge network layers. Finally, after the last decoder stage, the image features are classified into two classes, i.e., the plaque area and the background using the softmax classifier layer (pink). An efficient ADAM optimizer was used to reduce the cross-entropy loss in plaque segmentation. If y_i_ represents the GT label and $a_{i}$ means the softmax classifier probability, then the cross-entropy (*CE*) loss is described by Equation (1) as follows:

(1) $$L_{CE}=-{[(y}_{i}{\times \log\;a}_{i})+(1-y_{i})\;\times \;\log(1-a_{i})]$$

### 2.3. Experimental Protocol

We performed various experiments using both the Japanese and Hong Kong databases. Table 1 represents a consolidated list of the experiments with selected combinations of the databases. All experiments were conducted in the same programming environment (MATLAB 2019b) as well as with the same hardware configuration (core i7 8th Gen, 16 GB RAM, 8 GB NVIDIA Quadro P4000 Graphics processor) and operating system using Windows 10. Additionally, the same hyperparameters were applied to all experiments such as: batch size = 10, epochs = 100, optimizer = ADAM, learning rate = 1 × 10^−4^ Input grayscale image and binary mask image sizes were also fixed to 224 × 224 for all experiments.

#### 2.3.1. Unseen AI Data Experiments

In experiment#1, (Unseen AI-1 (Tr: JAP, Te: HK)), we trained the UNet model with 330 low-risk images from Japanese DB and the trained model was saved. Furthermore, we tested the model on 300 HK DB. During the experiment, an entire database of a single ethnic group was used for training and DB from other ethnic groups was used for testing. Experiment#2 (Unseen AI-2 (Tr: HK, Te: JAP)) is the reverse of experiment#1 (Unseen AI-1 (Tr: JAP, Te: HK)), in which we performed the training of the same network with 300 HK databases and testing on 330 Japanese databases. In both experiments, one database was used for training and the other was used for testing.

#### 2.3.2. Seen AI Data Experiments

Furthermore, we decided to check the same network with a mixture of both databases. Continuing with our hypothesis, in this experiment#3 (Seen AI-1 CV w/ Mixed), we mixed up 330 Japanese and 300 HK databases and used the 10-fold partition method to cross-validate each image. In the 10-fold cross-validation method, 90% of images of the mixed database were used as the training images and 10% of the images were used as the test images. Furthermore, 10% of the test images were swapped from the original database, and a fresh batch of 10% test images became available for testing. Likewise, 10 different sets of 10% images were used for testing; therefore, the experiment was repeated 10 times. Thus, in a 10-fold cross-validation, each image gets a chance to go for testing. 

Alongside the mixed database, we also checked the performance of the UNet model for individual databases by only cross-validating a single database. In experiment#4 (Seen AI-2 CV w/ JAP), we only used the Japanese DB and cross-validated all 330 images using the 10-fold cross-validations explained earlier. Similarly, in experiment #5 (Seen AI-3 CV w/ HK)), we performed 10-fold cross-validations on the 300-image Hong Kong DB. Table 2 show the summary of the experiments used in this study.

## 3. Results

**Figure 3:**
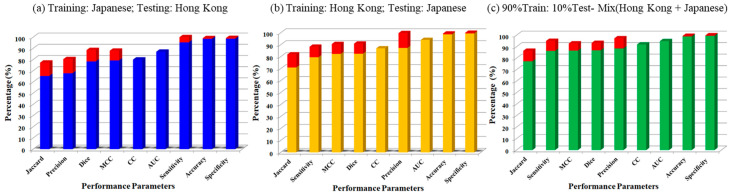
Bar chart for all performance parameters of Unseen AI and Seen AI experiments.

In this section, we will discuss the classification results of all experiments listed in Table 2. Table 2 contains a summary of the classification results of all experiments. The classification parameters of the UNet training model include (i) the correlation coefficient (CC); (ii) the area under the curve (AUC); (iii) accuracy; (iv) sensitivity; (v) specificity; (vi) precision; (vii) Mathew’s correlation coefficient; (viii) dice similarity coefficient (DSC); and (ix) the Jaccard index (JI). In the first experiment#1, i.e., (Unseen AI-1 (Tr: JAP, Te: HK)), the UNet model was trained with 330 Japanese DB images and tested on 300 Hong Kong DB images. Therefore, nine mean classification parameters’ values of the 300 HK DB images are 0.8, 0.87, 98.55, 95.41, 98.64, 67.82, 79.29, 78.38, and 65.42, respectively. Figure 3a shows the bar chart of the mean classification parameters of the Unseen AI-1 (Tr: JAP, Te: HK). Now, in experiment#2 (Unseen AI-2 (Tr: HK, Te: JAP)), the training and testing databases were swapped. The same model was trained on 300 HK DB images and tested on 330 Japanese DB. Therefore, with Unseen AI-2 (Tr: HK, Te: JAP), we achieved nine mean classification parameters’ values for the 330 Japanese DB images as 0.87, 0.94, 98.67, 79.52, 99.47, 87.29, 82.29, 82.49, and 70.98. Figure 3b shows the bar chart of the mean classification parameters of the Unseen AI-2 (Tr: HK, Te: JAP).

Now, in experiment #3, i.e., (Seen AI-1 CV w/ Mixed), the Japanese DB and Hong Kong DB was mixed and the combined DB was cross-validated using 10-fold cross-validations. Therefore, in this experiment using cross-validation, each image was tested once. The mean classification parameters’ values for the mixed database were 0.92, 0.95, 99.01, 86.37, 99.52, 88.55, 86.68, 86.89, and 77.34. Figure 3c shows the bar chart of the mean classification parameters of experiment #3, i.e., (Seen AI-1 CV w/ Mixed).

We also tested the monoethnic DB using the 10-fold cross-validation method. Using this strategy, we performed experiment#4 (Seen AI-2 CV w/ JAP) in which only 330 Japanese DB images were used for the 10-fold cross-validation. For this experiment, the mean classification parameters’ values are 0.87, 0.93, 98.99, 91.25, 99.26, 81.01, 84.88, 84.65, and 74.62. In an experiment similar to experiment#5 (Seen AI-3 CV w/ HK), only 300 HK DB images were used for 10-fold cross-validation. The mean classification parameters are 0.89, 0.95, 98.96, 87.27, 99.43, 86.50, 86.04, 86.29, and 76.59.

### Visual Segmentation Results

**Figure 4:**
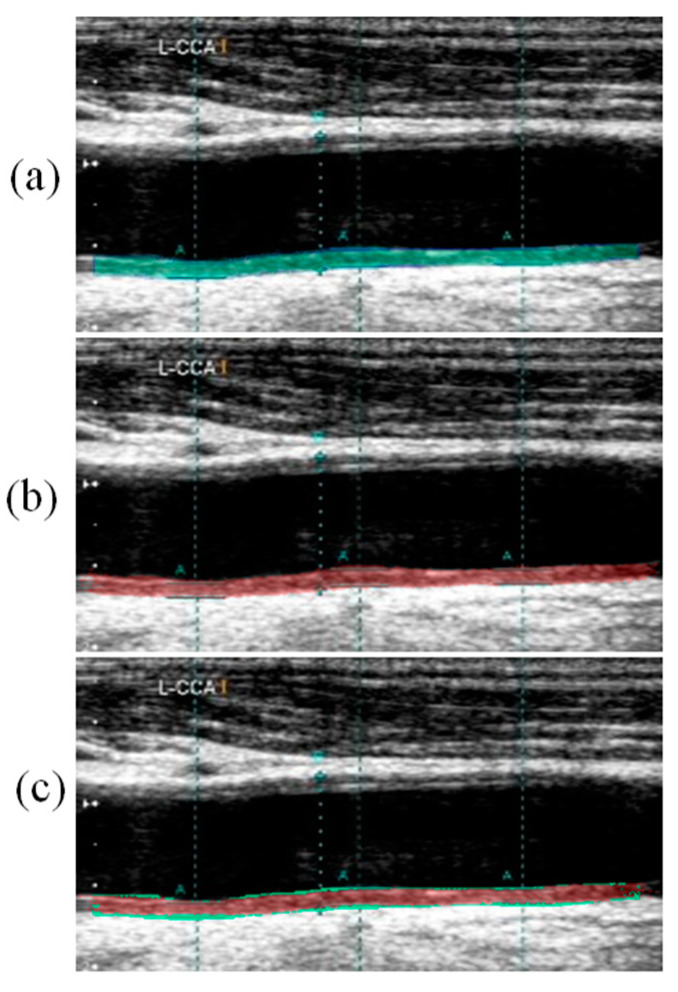
Generation of difference between the GT and AI plaque overlay images. (**a**) GT plaque overlay (**b**) AI-estimated plaque overlay (**c**) Difference between GT and AI plaque overlay image.

Figure 4 below shows the generation of difference between and the GT images and AI segmented images. Figure 4a shows the overlay of the GT mask and raw image in green color. Figure 4b shows the overlay of the AI-segmented mask and raw image in red color. Figure 4c shows the overlay of difference between the GT and AI segmented images. Thus, in Figure 4c, the red color shows the AI predicted plaque area whereas the green color represents the difference plaque area.

**Figure 5:**
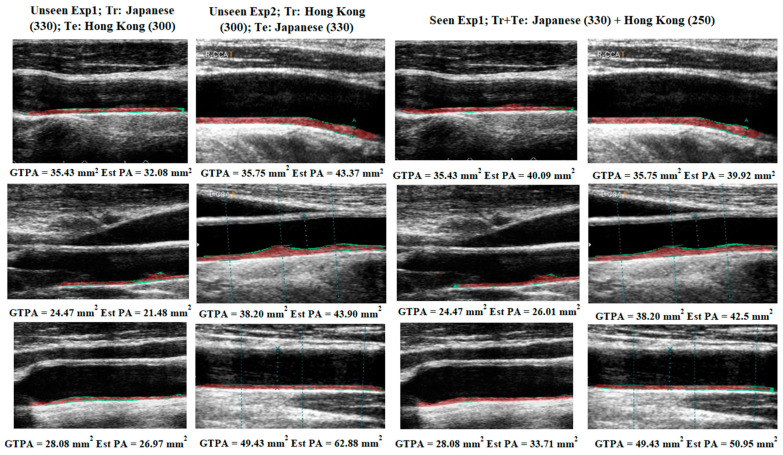
Visual results of the Unseen AI and Seen AI experiments. [Column 1 shows the absolute difference images of the HK test DB for experiment#1 (Unseen AI-1 (Tr: JAP, Te: HK)). Similarly, Column 2 shows the absolute difference images of the Japanese test DB for experiment#2 (Unseen AI-2 (Tr: HK, Te: JAP)). However, columns 3 and 4 represent the difference images from the mixed database of experiment#3 (Seen AI-1 CV w/ Mixed)].

Figure 5 below shows the absolute difference image of the UNet model output (red) and GT mask overlay on the grayscale image (green).

## 4. Performance Evaluation

We performed various performance evaluations and statistical tests on the test data to validate our hypothesis. We calculated the mean of all performance and statistical parameters for both DL models and presented them in Table 3.

### 4.1. Correlation between AI Models and Ground Truth 

**Figure 6:**
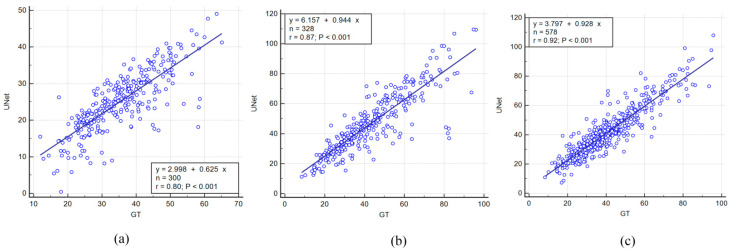
Regression plots for Unseen AI and Seen AI experiments. (**a**) Experiment #1 (Unseen AI-1 (Tr: JAP, Te: HK)) (**b**) experiment #2 (Unseen AI-2 (Tr: HK, Te: JAP)) (**c**) experiment #3 (Seen AI-1 CV w/ Mixed).

The correlation coefficient (CC) is an effective statistical parameter used to analyze the relationship between two quantities. It ranges between ”0” and “1”, representing a degree of match between the two quantities. A high value (close to “1”) represents a high match, whereas a low value represents a low match. Table 3 shows the CC value for all experiments. Additionally, Figure 6a–c below show the CC values and regression curve for experiments #1, #2, and #3. As depicted in the figure, the CC between UNet and GT for experiment #1 (Unseen AI-1 (Tr: JAP, Te: HK)) is 0.8, 0.87 for experiment #2 (Unseen AI-2 (Tr: HK, Te: JAP)), and 0.92 for experiment #3 (Seen AI-1 CV w/ Mixed)—all having *p* < 0.001. Additionally, the CC for exp#4 (Seen AI-2 CV w/ JAP) is 0.87, and 0.89 for experiment #5 (Seen AI-3 CV w/ HK).

### 4.2. Receiver Operating Characteristics and AUC

**Figure 7:**
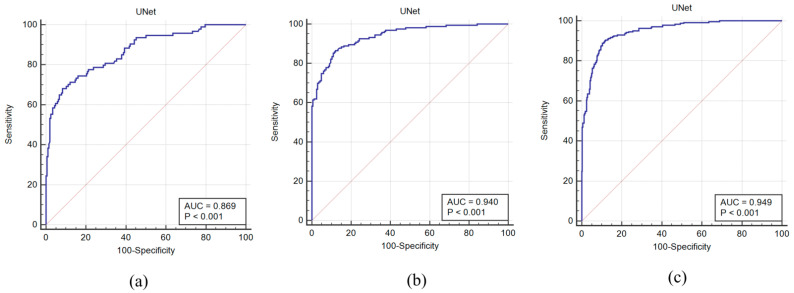
Receiver operating characteristics (ROC) curves and AUCs for the Unseen AI and Seen AI experiments. (**a**) Experiment #1 (Unseen AI-1 (Tr: JAP, Te: HK)) (**b**) experiment #2 (Unseen AI-2 (Tr: HK, Te: JAP)) (**c**) experiment #3 (Seen AI-1 CV w/ Mixed).

The receiver operating characteristics (ROC) curve and the area under the ROC curve are important performance parameters in medical image analysis. We used the ground-truth plaque area (GTPA) threshold value of 40 mm^2^ to generate the GT binary labels “1” for high-risk images and “0” for low-risk images. Furthermore, the deep learning plaque area (DLPA) was used to plot the ROC curve between the GTPA labels and DLPA scores. Figure 7a–c show the ROC curves and AUC values for experiment #1 (Unseen AI-1 (Tr: JAP, Te: HK)), experiment #2 (Unseen AI-2 (Tr: HK, Te: JAP)), and experiment #3 (Seen AI-1 CV w/ Mixed).

### 4.3. Bland–Altman Plots

**Figure 8:**
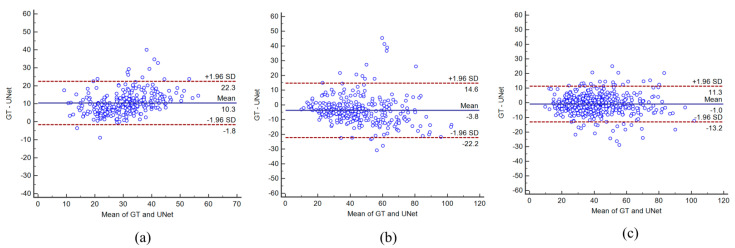
Bland-Altman’s plots for Unseen and Seen experiments. (**a**) Experiment #1 (Unseen AI-1 (Tr: JAP, Te: HK)) (**b**) experiment #2 (Unseen AI-2 (Tr: HK, Te: JAP)) (**c**) experiment #3 (Seen AI-1 CV w/ Mixed).

Bland–Altman’s plots between the UNet-GT for experiment #1 (Unseen AI-1 (Tr: JAP, Te: HK)), experiment #2 (Unseen AI-2 (Tr: HK, Te: JAP)), and experiment #3 (Seen AI-1 CV w/ Mixed) are shown in Figure 8a–c. From the plots, the Seen AI experiment #1 model’s output is closely concentrated along the central lines.

### 4.4. Paired Sample t-Test and ANOVA Test

**Figure 9:**
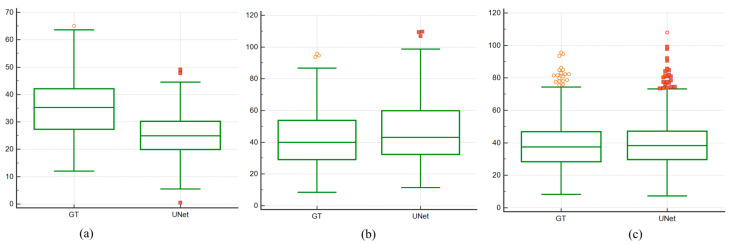
Comparison of paired *t*-test curves for Unseen AI and Seen AI database experiments. (**a**) Experiment #1 (Unseen AI-1 (Tr: JAP, Te: HK)) (**b**) experiment #2 (Unseen AI-2 (Tr: HK, Te: JAP)) (**c**) experiment #3 (Seen AI-1 CV w/ Mixed).

**Figure 10:**
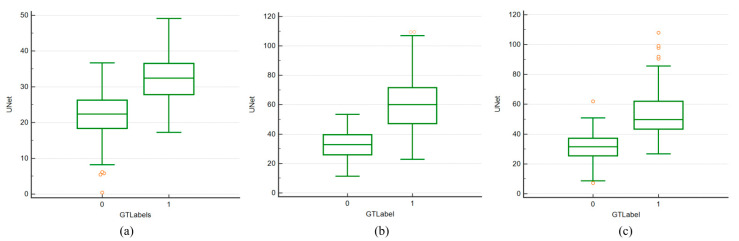
Comparison of ANOVA test curves for Unseen AI and Seen AI database experiments. (**a**) Experiment #1 (Unseen AI-1 (Tr: JAP, Te: HK)) (**b**) experiment #2 (Unseen AI-2 (Tr: HK, Te: JAP)) (**c**) experiment #3 (Seen AI-1 CV w/ Mixed).

Paired Sample *t*-test: The box and whiskers plot is a convenient way to show the data distribution. Figure 9a–c below show the paired sample *t*-tests between the UNet estimated area and GT area using a box and whiskers plot for experiments #1, 2, and 3, respectively. As the boxes in the plot show, for experiment #3 (Seen AI-1 CV w/ Mixed), the median values of UNet are close to the GT median values compared to other experiments. Figure 10a–c show the box and whiskers plot for the analysis of variance (ANOVA) test between the UNet and GT area for all three experiments: experiment #1 (Unseen AI-1 (Tr: JAP, Te: HK)), experiment #2 (Unseen AI-2 (Tr: HK, Te: JAP)), and experiment #3 (Seen AI-1 CV w/ Mixed).

### 4.5. Figure of Merit

The figure of merit (FoM) is defined in terms of the central tendency of the error. Let $a_{ai}\left(n\right)$ and $a_{gt}$ represent the plaque area for the UNet model and GT, respectively, for image “*n*”. Considering *N* as the total number of scans, the corresponding mean area ${\bar{a}}_{ai}\left(e\right)$ for the experiment “*e*” and for GT ${\bar{a}}_{gt}$ and then the FoM can be expressed as Equation (2):

(2) $$FoM\left(e\right)=100-\left[\left(\frac{\left|{\bar{a}}_{ai}\left(e\right)-{\bar{a}}_{gt}\right|}{{\bar{a}}_{gt}}\right)*100\right]$$

Thus, the FoM for experiment #1 (Unseen AI-1 (Tr: JAP, Te: HK)), experiment #2 (Unseen AI-2 (Tr: HK, Te: JAP)), experiment #3 (Seen AI-1 CV w/ Mixed), experiment #4 (Seen AI-2 CV w/ JAP), and experiment #5 (Seen AI-3 CV w/ HK) was calculated using the above method to be 70.96%, 91.14%, 97.57%, 88.89%, and 99.14%, respectively.

## 5. Discussion

**Figure 11:**
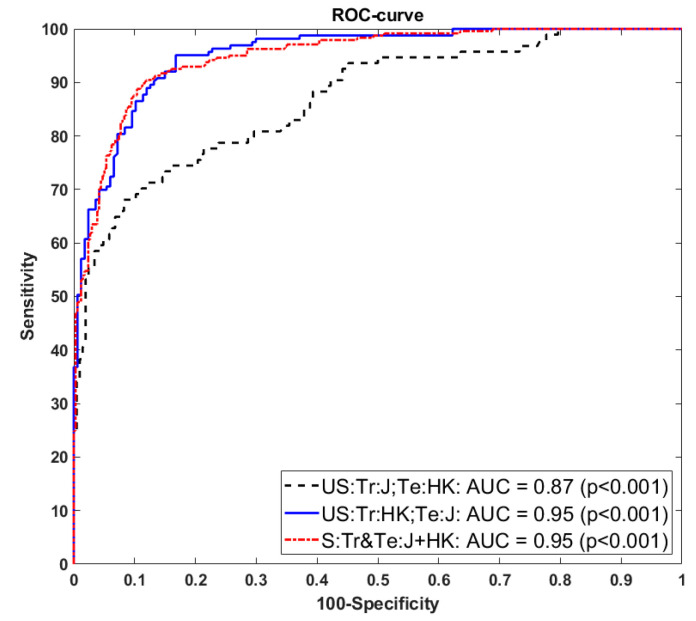
Comparison of ROC curve for unseen and seen database experiments.

We presented herein the Unseen AI-based deep learning system for the segmentation of carotid B-mode plaque images. The system used two ethnic databases, i.e., the low-plaque Japanese diabetic database and the Hong Kong database. Furthermore, we hypothesized that the system is able to perform well with Unseen AI data. To prove our hypothesis, we performed a series of experiments as shown in Table 1. One unseen experiment used one ethnic DB for training and the other used another ethnic DB for testing and the second experiment did the opposite. The third experiment used a mix of the Japanese and Hong Kong databases for training and testing. In this Seen AI experiment, a cross-validation approach was used to test each image from the mixed database. However, in this mixed data experiment, the training and testing images were from the same pool of the mixed database. We also extended our work to experiments #4 and #5, which performed the cross-validation of same ethnic databases on the Japanese and Hong Kong databases. A comparison of the ROC curves for experiments #1, 2, and 3 is shown in Figure 11. Comparing the three seen and two unseen experiments, we can conclude that the system can segment a low atherosclerotic plaque from the unseen database. Additionally, the differences between unseen and seen experiments shown in Table 4 are within the range of 15%.

### 5.1. Benchmarking

Table 5 shows the benchmarking studies that involve the multi-ethnic databases for atherosclerotic plaque measurement. Modern techniques for the segmentation of the wall plaque in carotid B-mode ultrasound have been active since 2010. Even though the ultimate goal of these techniques is cIMT measurement, we briefly discuss them here. Most of the methods were developed by Suri’s group from AtheroPoint™ (Roseville, CA, USA) [52,53]. In the early start-up methods for wall segmentation, the first-order absolute moment (FOAM) method for completely automated local statistical-based method (CALSFORM) was shown [54,55,56]. The region of interest detection was important for automated LI/MA detection. Three methods were discussed for ROI estimation in which the plaque wall was supposed to be detected [57]. Scale-space methods were invented, which had the flexibility of automatically finding the LI and MA borders, which proved to be more robust [29,30]. These techniques are also summarized in the review by Molinari et al. [58]. This system was then commercialized into AtheroEdge™ 1.0 and applied for a multicenter clinical study. A review was published comparing the different methods [59]. Several validation methods and applications were also developed [60,61]. All the above studies were focused on wall segmentation and cIMT measurement. Recently, AI tools were developed for the joint estimation of wall thickness and area estimation by Biswas et al. [10]. Molinari et al. used five ethnic databases for IMT measurement using automated and semi-automated methods [15]. However, their systems did not involve deep-learning methods. Mean ± SD for IMT measurement for all these methods were 0.811 ± 0.292 (CALEX), 0.779 ± 0.264 (CARES), 0.806 ± 0.294 (CAMES), 0.873 ± 0.323 (CAUDLES-EF), and 0.786 ± 0.251 (FOAM), respectively (in mm). Ikeda et al. used three ethnic groups’ (Japanese, Italy and Hong Kong) data for IMT measurement at the bulb area [62]. Another group of Zhou et al. [47] presented a deep learning-based method for segmentation of atherosclerotic plaque from carotid ultrasound images. Their system used two ethnic databases: namely those of Stroke Prevention and Atherosclerosis Research Centre (SPARC), from London, Canada; and Chinese data from Zhongnan Hospital (Wuhan, China). However, their work does not focus on the effect of unseen databases on the system’s performance. In a multi-ethnic atherosclerosis study, Carol et al. 2018 used four ethnic people, i.e., White, Chinese, Black, and Hispanic people from across the country [63]. Purpose of their study was to analyze the relation between TPA and CVD risk factors in middle age group. Recently, by the team of Suri, Jamthikar et al. in 2020 proposed a Framingham risk score-based model for stroke risk assessment. They included 648 patients from three ethnic databases, and both left and right carotid artery images of each patient were included in the experiment. A 10-year risk prediction model, atherosclerosis CVD (ASCVD) developed by American College of Cardiology/American Heart Association, was used in their research. Furthermore, we compared our system with these studies, and found that our study has been solely focused upon the effect of ethnicity on the performance of the system [64].

### 5.2. Short Note on Image Quality

Image quality in medical imaging such as MRI [65], CT [66], X-ray [67], and US [68] certainly plays an important point during the design of the computer-aided diagnosis (CAD) system. This is even more crucial during the US CAD design, since the quality of the images are subjected to several factors such as (i) strong contact between the probe and the skin; (ii) the gel used at the contact point; (iii) the gain control systems of the scanner; and (iv) the role of the harmonic imaging system and compound imaging systems were on during the beam formation and scanning process. 

There have been several CAD systems which directly employ denoising solutions for US scans [13,69] or scale-space filtering methods [70]. One way to handle the denoising process is to compute the signal-to-noise ratio (SNR) or contrast-to-noise ratio (CNR) of US scans [71] and if they are under threshold limits, they can then be used for deep learning solutions. Note that these threshold limits are partially dependent on the datasets. Not all datasets need denoising solutions. Lastly, the latest US scanners have started to produce high-resolution images. Old legacy machines certainly need special denoisers for removing salt and pepper noise, speckle noise, or even getting rid of black shadows.

In our study, the images were selected by the experienced sonographer and filtered out based on their judgement of image quality to characterize the plaque area. Thus, we never encountered images which had low image quality. One can, however, design a method which can add denoisers in the loss function for improvements in the plaque detection process. This is similar to the approach of adding the penalty function in the boundary segmentation models [72] or using a partial differential equation-based smoothing process [73].

In our current study, we used two types of databases, namely the Japanese and Hong Kong ones. The Hong Kong database images are much nosier compared to the Japanese database images. The major objective of this paper was to address the use of different databases and prepare such a deep learning model which can train on one database and predict on other databases. We also swapped the databases and each time the accuracy was above the threshold mark. However, the images which were low in quality or the sonographer’s lack of experience were not considered in our study.

### 5.3. Strength, Limitations and Future Extensions

As seen in Table 4, the percentage difference between the seen and unseen data experiments suggests that the system can perform atherosclerotic plaque segmentation. The system demonstrated significant results with unseen data experiments. Additionally, the system is able to capture the plaque data in the low-contrast images of the Hong Kong ethnic database. Furthermore, the system performs the testing of images in almost real-time. A major limitation of our system was the inclusion of the noisy Hong Kong database in the system. However, with the inclusion of some noise suppression methods [69], performance can be improved. Furthermore, (i) the system can be extended and applied for the segmentation of temporal data and (ii) we can characterize the segmented plaque wall using classification methods [74]. Multi-modality validation can also be achieved using joint ultrasound and CT [75]. 

The loss function plays an important role during the segmentation and classification paradigm [46]. In our recent publication, we tried to apply the dice loss function to stroke publications [37,76]. This was attempted for heavy plaque images having a partial volume effect (partial plaque available in the plaque zones of some pixel locations). The results were very encouraging. In our current study, most of the arteries were low plaque and mainly straight due to the nature of the common carotid artery. Thus, the identification of the region-of-interest was very straight-forward in the patient test data. There is no such issue of partial volume because the media region clearly showed a high-intensity zone. Thus, the simple cross-entropy function was successful in demonstrating our hypothesis of “Unseen AI”. However, we will pursue other loss functions in the future such as MSE and DSC loss functions.

## 6. Conclusions

This was the first pilot study in the area of carotid ultrasound-based imaging that used two different ethnic groups in the AI framework, demonstrating the concept of “Unseen AI”. Two distinct cohorts, i.e., the Japanese and Hong Kong ones, were used to prove the hypothesis that “Unseen AI” was close (<10%) to “Seen AI”. The study presented a four-layer deep learning UNet architecture for atherosclerotic plaque wall segmentation in the common carotid arteries. Our performance parameters such as mean accuracy, dice-similarity, and correlation-coefficient were 98.55, 78.38, and 0.80 (*p* < 0.0001), respectively, when using the Unseen AI pair-1 with the Japanese database for training and the Hong Kong database for testing. The same parameters were 98.67, 82.49, and 0.87 (*p* < 0.0001), respectively, when using the Unseen AI pair-2 consisting of Hong Kong training and Japanese testing. When benchmarking against the Seen AI, using the cross-validation protocol, for a mixed cohort (Japanese and Hong Kong), our system demonstrated the same parameters to be 99.01, 86.89, and 0.92 (*p* < 0.0001), respectively, validating our hypothesis and stability of the system. We further concluded that an online system takes less than one second, and such a system can be extended to other deep learning and hybrid deep learning models.

## Figures and Tables

**Table 1:** Summary of all experiments performed on a multiethnic database.

Exp #	Name of Exp	Training DB	Testing DB	Training Protocol
Exp #1	Unseen AI-1 (Tr: JAP, Te: HK)	Japanese 330	Hong Kong 300	All Japanese DB for training
Exp #2	Unseen AI-2 (Tr: HK, Te: JAP)	Hong Kong 300	Japanese 330	All Hong Kong DB for training
Exp #3	Seen AI-1; CV w/ Mixed	Japanese (330) + Hong Kong (250)	Japanese (330) + Hong Kong (250)	10-fold cross-validation
Exp #4	Seen AI-2; CV w/ JAP	Japanese (330)	Japanese (330)	10-fold cross-validation
Exp #5	Seen AI-3; CV w/ HK	Hong Kong (300)	Hong Kong (300)	10-fold cross-validation

CV w/: cross-validation with; JAP: Japanese; HK: Hong Kong.

**Table 2:** Classification parameters of the test dataset of all experiments.

Experiment #	UNet Experiments	ACC	Sens	Spec	Prec	MCC	DSC	JI
Exp #1	Unseen AI-1 (Tr: JAP, Te: HK)	98.55 ± 0.57	95.41 ± 5.29	98.64 ± 0.62	67.82 ± 12.55	79.29 ± 8.64	78.38 ± 10.11	65.42 ± 11.84
Exp #2	Unseen AI-2 (Tr: HK, Te: JAP)	98.67 ± 0.67	79.52 ± 8.84	99.47 ± 0.67	87.29 ± 12.45	82.29 ± 8.34	82.49 ± 8.44	70.98 ± 10.90
Exp #3	Seen AI-1 CV w/ Mixed	99.01 ± 0.44	86.37 ± 8.69	99.52 ± 0.41	88.55 ± 8.82	86.68 ± 6.19	86.89 ± 6.43	77.34 ± 9.15
Exp #4	Seen AI-2 CV w/ JAP	98.99 ± 0.58	91.25 ± 8.13	99.26 ± 0.64	81.01 ± 14.80	84.88 ± 9.49	84.65 ± 10.68	74.62 ± 13.54
Exp #5	Seen AI-3 CV w/ HK	98.96 ± 0.39	87.27 ± 7.70	99.43 ± 0.42	86.50 ± 10.45	86.04 ± 7.72	86.29 ± 8.31	76.59 ± 9.96

ACC: accuracy; Sens: sensitivity; Spec: specificity; Prec: precision; MCC: Mathew’s correlation coefficient; DSC: dice similarity coefficient; JI: Jaccard index.

**Table 3:** Performance and statistical parameters of the test dataset of all experiments.

Experiment #	UNet Experiment	CC	AUC	FoM
Exp #1	Unseen AI-1 (Tr: JAP, Te: HK)	0.8	0.87	70.96
Exp #2	Unseen AI-2 (Tr: HK, Te: JAP)	0.87	0.94	91.14
Exp #3	Seen AI-1, CV w/ Mixed	0.92	0.95	97.57
Exp #4	Seen AI-2, CV w/ JAP	0.87	0.93	88.89
Exp #5	Seen AI-3, CV w/ HK	0.89	0.95	99.14

CC: correlation coefficient; AUC: area under the curve; FoM: figure of merit.

**Table 4:** Comparison of experiments and validation of hypothesis.

#Exp	Comparison of Experiments	CC	AUC	ACC	Sens	Spec	Prec	MCC	DSC	JI
3-1	Seen AI-1 CV w/ Mixed-Unseen AI-1 (Tr: JAP, Te: HK)	13.04	8.42	0.46	−10.47	0.88	23.41	8.53	9.79	15.41
		**~**	**√**	**√**	**√**	**√**	**~**	**√**	**√**	**~**
3-2	Seen AI-1 CV w/ Mixed-Unseen AI-2 (Tr: HK, Te: JAP)	5.43	1.05	0.34	7.93	0.05	1.42	5.06	5.06	8.22
		**√**	**√**	**√**	**√**	**√**	**√**	**√**	**√**	**√**
4-1	Seen AI-2, CV w/ JAP-Unseen AI-1 (Tr: JAP, Te: HK)	8.05	6.45	0.44	−4.56	0.62	16.28	6.59	7.41	12.33
		**√**	**√**	**√**	**√**	**√**	**~**	**√**	**√**	**~**
4-2	Seen AI-2, CV w/ JAP-Unseen AI-2 (Tr: HK, Te: JAP)	0.00	−1.08	0.32	12.85	−0.21	−7.75	3.05	2.55	4.88
		**√**	**√**	**√**	**~**	**√**	**√**	**√**	**√**	**√**
5-1	Seen AI-3, CV w/ H -Unseen AI-1 (Tr: JAP, Te: HK)	10.11	8.42	0.41	−9.33	0.79	21.60	7.85	9.17	14.58
		**√**	**√**	**√**	**√**	**√**	**~**	**√**	**√**	**~**
5-2	Seen AI-3, CV w/ H -Unseen AI-2 (Tr: HK, Te: JAP)	2.25	1.05	0.29	8.88	−0.04	−0.91	4.36	4.40	7.32
		**√**	**√**	**√**	**√**	**√**	**√**	**√**	**√**	**√**
3-4	Seen AI-1 CV w/ Mixed-Seen AI-2, CV w/ JAP	5.43	2.11	0.02	−5.65	0.26	8.51	2.08	2.58	3.52
		**√**	**√**	**√**	**√**	**√**	**√**	**√**	**√**	**√**
3-5	Seen AI-1 CV w/ Mixed-Seen AI-3, CV w/ HK	3.26	0.00	0.05	−1.04	0.09	2.32	0.74	0.69	0.97
		**√**	**√**	**√**	**√**	**√**	**√**	**√**	**√**	**√**

**Table 5:** Benchmarking table showing multiethnic database studies for atherosclerotic plaque measurement.

Sr#	Authors and Year	Cohorts	Images	Purpose	Model
1	Molinari et al., 2012 [15]	Torino (n1) Nicosia (n2) Cagliari (n3) Porto (n4) Hong Kong (n5)	n1 = 200 n2 = 100 n3 = 42 n4 = 23 n5 = 300	IMT measurement using auto and semi-auto methods	ML
2	Ikeda et al., 2013 [62]	Japanese (n1) Italy (n2) Hong Kong (n3)	n1 = 259 n2 = 98 n3 = 300	IMT measurement in Bulb area	ML
3	Zhou et al., 2020 [47]	SPARC (n1) Chinese * (n2)	n1 = 510 n2 = 638	Plaque area measurement in ICA and CCA images	DL
4	Carol et al., 2018 [63]	White (n1) Chinese (n2) Black (n3) Hispanic (n4)	n1 = 946 n2 = 185 n3 = 595 n4 = 479	Carotid plaque analysis using manual method	Statistical method
5	Jamathikar et al., 2020 [64]	Japanese (n1) Asian-Indian (n2) Spanish (n3)	n1 = 404 n2 = 628 n3 = 264	Framingham risk score-based stroke risk stratification	ML
6	Proposed method	Japanese (n1) Hong Kong (n2)	n1 = 330 n2 = 300	Plaque area measurement in CCA images	DL

SPARC: Stroke Prevention and Atherosclerosis Research Centre, London, Canada; * Zhongnan Hospital (Wuhan, China).

## Data Availability

The Institutional Review Board has been issued to AtheroPoint, Roseville, CA, USA and therefore this database cannot be shared publically.
